# ‘Virtual’ Action Research: Two examples

**DOI:** 10.1007/s11213-022-09620-0

**Published:** 2022-11-09

**Authors:** Frank Stowell, Pavla Kramarova

**Affiliations:** 1 Emeritus Professor Systems and Information Systems, School of Computing, University of Portsmouth, Portsmouth, UK; 2 Portsmouth Business School, University of Portsmouth, Portsmouth, UK

**Keywords:** Soft Action Research; Virtual Action Research, Soft Systems, AIM

## Abstract

Action research is the predominant approach for organisational inquiry, but it comes in many guises and in some cases ends up as all action and no research. A common feature of all action research is the necessity to meet stakeholders in person in order to gain an understanding ‘from within’, this is particularly the case where ‘soft’ action research is the basis of the inquiry. In this paper first, we reflect, briefly, upon the history of action research and second, upon the lessons leant from the outcomes from two projects. The first project was conducted ‘virtually’, between a college in the USA and one in the UK and the second undertaken during the disruption caused by the restrictions arising from the Covid-19 pandemic. The experiences gained from these two ‘virtual’ action research projects and the lessons learnt are of interest to both the ‘traditional’ and ‘soft’ action research community.

## Introduction

This paper is a natural successor to the Stowell and Cooray paper in which they explored the possibility of undertaking ‘Virtual Action Research for Virtual Organisations’, (Stowell and Cooray 2016). In the paper they described how discussions between two academic institutions took place several thousand miles apart. The discussion concerned improving the operation of the international programme’s between a College in the USA and a University in the UK. Communication between the two groups was using the free web based synchronous software tool ‘Wiggio’ that provided audio, video and texting capabilities to enable participants to communicate synchronously. The method of investigation selected for this study was the Appreciative Inquiry Method (AIM). AIM is easily learnt by participants and ideal in this situation where participants are thousands of miles apart. Starting with a simple Venn diagram means it can be easily drawn on a computer and ideal where the question to investigate has already been agreed by participants. The first stage begins with each participant producing their own Venn diagram, which provide the basis for discussion and development of a shared understanding. The outcome of this research provided many useful lessons and signposted a way of undertaking action research (A/R) virtually. Although this approach to A/R offered many possibilities it was the Government restrictions on face-to-face engagement caused by the Covid-19 pandemic of 2019–2020 that created a new challenge and paradoxically, provided another opportunity to develop further the application of AIM as a virtual method for A/R. Whereas Stowell and Cooray’s project was initiated by the need to communicate with partners separated by an ocean, the Covid 19 restrictions Kramarova and Stowell faced meant that physical proximity between individuals could not take place. Other ways of undertaking A/R had to be developed. It was logical to develop the lessons learnt from Stowell and Cooray’s research to explore the possibility of using these ideas where no physical contact between participants was possible. The experiences gained provides valuable lessons for other such projects and expand the reach of A/R beyond physical boundaries.

In this paper we begin by summarising the method of action research within the context of organisational inquiry followed by what we term soft action research (Stowell and Cooray 2017, p.121). This is followed by the outcomes of the Stowell and Cooray paper then we provide some examples from the Kramarova and Stowell (2021a,b,c) field study where AIM was used as the basis of a virtual soft action research study.

## Organisational Inquiry

In an early, but ground breaking paper, Checkland (1985, p.765) drew attention to the pervading methods of enquiry dominating Operational Research (O/R) and Systems Engineering (S/E). Approaches inquiring into, what Checkland calls in this early paper, human activity systems. He argued that we should seek to view a situation as a whole rather than think of it as something that can be formulated into a model from which an optimum outcome can be derived. In this paper we describe two such applications that helped to explore the idea of virtual action research further. To do this we will first provide an overview of action research and the intellectual basis on which it is based. This is followed by a brief discussion of one project in which soft A/R was used and then specific examples from a second project where soft action research was conducted entirely in a virtual setting. This is followed by an account of some of the key lessons learnt from both projects.

## Organisational Inquiry and Action Research

Thinking in terms of a fixed model or a ‘solution’ based upon an ‘ideal’ case from past success is deficient.^1^ To paraphrase Susman and Evered (1978) we can say that while models provide the theorists with a means of depicting organisational behaviour, ‘real world’ experience of applying these models is not always as successful as the models predicted. This is because the measurement of success between theory and practice are rarely the same, particularly if the theory is not enriched by the lessons from the practice.

The difficulty reductionist models have is that social groups are complex and do not always behave as models predict, exposing the division between practice and theory. It was the realisation that problems could not be solved by theory alone that was the motivation behind Action Research.^2^ Lewin (1948), believed that a situation should be considered as a ‘whole’, those that make it up are influenced not just by the situation itself but by a variety of life experiences.

In their 1978 paper Susman and Evered write ‘…*the crisis in organisational science is reflected in a conception of social facts that can be drawn on by practitioners when they are ready to apply them. This conception encourages a separation of theory from practice because published research is read more by producers of research than practitioners…*’. Their claim is that positivist models dominate organisational inquiry and ‘…*may only inadvertently serve and sometimes undermine the values of organisational members…*’ (ibid pps.582–583). Each situation is unique and as such any investigation has to consider alternative ways to gain understanding. Individuals that make it up are there for different reasons, which are, as Vickers^3^observed, constantly revised or confirmed and as such will have different meanings for each person. As Lewin pointed out those that make up an organisation may not share the same cultural experiences, business ethos or even language. In a summary of action research approaches Hart says ‘…*the importance of context and the organisational perspective means taking into account different levels and interactions to be considered in the appreciative setting* (2013, p.58). This observation is heightened by the evolution of wireless communications where those that make up an organisation may be remote, may not share the same language, culture or even the same continent (Stowell and Cooray 2017, p.122).

Undertaking organisational inquiry has always been challenging and what we take as Action Research now comes under many guises and Lewin’s idea has developed since his original concept and has produced several interpretations. Many of these have yielded valuable insights into an organisational inquiry. Bradbury et al (2008, pp.77–92) provide one useful account of the subsequent development of these ideas across disciplines and from four different perspectives. Bradbury^4^ traces the development of A/R from the Socio technical perspective of the Tavistock Institute, Organisation Development, Community building and Somatic development and Organisation Development. The Reason and Bradbury (2013) text on A/R provides many examples of the practice and theory of A/R but it does not tell the whole story. Aside from a passing reference by Ison (2013, pp.139–158) there is no mention of the 30-year pioneering work at the university of Lancaster’s Action Research programme nor acknowledgement of Vickers notion of Appreciative Inquiry Systems (e.g. 1968 (a)) despite references to Cooperrider and Appreciative Inquiry; important omissions. My point is not a criticism of Reasons text, which makes a valuable contribution to the literature, but to highlight that not all approaches to A/R are covered. We make this observation because organisational inquiry is dependent upon the concept of ‘organisation’ taken by the researcher/analyst (e.g. Stowell 2021a) and embarking upon A/R will be directly influenced by this perception.

There are, as Bradbury has pointed out, different paradigms underpinning A/R, but it is not easy to place them into a coherent group. We find the summation by Collis and Hussey (2003), provides a useful context from which to think about A/R. They suggest three kinds of action research. Although not beyond criticism their categorisation does provide a general framework of approaches. These are;

Positivist approach to action research, also known as ‘classical action research’ perceives research as a social experiment. Accordingly, action research is accepted as a method to test hypotheses in a real-world environment.

Interpretive action research, also known as ‘contemporary action research,’ perceives business reality as socially constructed and focuses on specifications of local and organisational factors when conducting the action research.

Critical action research is a specific type of action research that adopts a critical approach towards business processes and aims for improvements.

To these we would like to add;

Soft Action Research. It is Based upon soft systems thinking and practice (Checkland 1999; Stowell 2021c). Each situation is approached without regard to previous experience (Epoché) the research is undertaken by the inquirer becoming a part of the situation of interest (A). At its core is the generation of a cycle of learning through which the participants can develop actions for change. The method used should be ‘agnostic’ to the situation (M) but has a synergistic relationship with the framework of ideas (F). The participation of stakeholders is central to soft systems thinking and action research’ (see Flood, 2010. pp.270–284). The criterion for soft A/R is recoverability; the activity should be made explicit to provide an outside observer with a ‘trail’ to all them to ‘recover’ the whole process. (Checkland and Holwell 1998, pp.9–11).

It is to the latter we now turn.

## Soft A/R

*Soft* A/R describes an approach to inquiry that is based on the Lancaster notion of ‘Systems’. Soft systems is underpinned by the phenomenology of Husserl and the Sociology of Schutz that were combined with the experience of theorists and practitioners such as Vickers 1983, West 1971 and Ackoff 1977. These ideas produced a whole new way of thinking about systems and of ‘organisational’ inquiry in particular (see Checkland 1999). The notion of ‘system’ now reflected an acceptance of the unpredictability of what is perceived, but what is perceived is transitory and shaped by experience. In this sense there is no absolute definition of a ‘system’ (of any kind). It is a particular phenomenon selected by us, (intentionality) and formed from a priori forms of experience. With this in mind we can say that the notion of ‘system’, anchored to the idea of holism, is that any ‘phenomenon’ we perceive is one selected from ‘something else’ that may exist in a physical sense but also possess a sensory existence which appears to different observers in different ways. We must ask ‘*what it is’;* what is its essence^5^ if we are to avoid repeating past mistakes.

Thinking in ‘systemic terms’ helps to gain a greater understanding of the ‘system’ of interest, and.

thinking about something as a System in this way gives ‘shape’ to the phenomenon with which we are concerned. It helps the observer to gain an appreciation of the situation in its entirety. In soft action research the inquirer becomes immersed in the situation, but as Checkland points out one of the dangers of action research is that it can decline *‘…into all action and no research.…and…it is difficult to see outcomes as any more than being anecdotal* (Checkland and Holwell 1998, p.14). *Soft* A/R should have a degree of structure, but one that does not constrain the participants. Any method employed by soft A/R should be in keeping with the underpinning paradigm and act as a guide, but not a set of rules to be slavishly followed.

The inquirer should attempt to ‘detach’ themselves from past experience^6^ but in any inquiry the inquirer should be aware of their own role and of their relationship with the participants. In SSM this is addressed through Analysis 1, 2 and 3, (Checkland and Poulter 2006, pp.27–38). Within SSM is a wide interpretation of the notion of Weltanschauung^7^ which Checkland says ‘…*is the most important concept in understanding the complexity of human situations, and indeed, the nature and form of SSM.*’ (ibid p.6).^8^In my view this observation underlines the ‘essence’ of soft systems itself (of which SSM is a practical manifestation). It is the acceptance of ‘subjectivity’. The implication of this is that *soft* Systems research accepts a world based upon the subjective experience of individuals. Everything that ‘exists’ is the result of personal experience which shapes how we behave. We learn from our experience of our ‘surrounding world’, the Umwelt, which is meaningful in a specific way for each of us. The meaning we attach to something or to an event is created from the culture or social setting in which it exists and the language we use to explain this is *abstract*. Each situation we encounter is ‘shaped’ by the way we perceive the world. In some respects, this is encapsulated in Vickers notion of the ‘Appreciative System’, which he describes as a unique interpretive screen that provides one amongst several ways of interpreting our experience (Vickers 1983, p.69).

Accepting the subjectivity of our experiences yet being able to ‘find out’ is not a trivial undertaking. The method of inquiry can shape the outcome, indeed even the selection of the ‘tools’ to be used can themselves have meaning for the inquirer. To this end the notion of FMA suggested by Checkland offers a way of reflecting upon the coherence of the inquiry. FMA in practice is as follows;

- ‘A’ Area of Interest. Although this appears to be straightforward care should be taken to be clear about the boundary as, without it, it is easy to find the inquiry gets out of control^9^
- ‘F’ Framework of ideas. To what intellectual perspective do you subscribe – i.e. where do you think you stand in the ‘intellectual universe’?
- ‘M’ Method employed. This is important and is often overlooked. M and F should have a synergistic discernible relationship.

## Selecting the Method of inquiry

The first task of the soft systems inquirer/facilitator is to create an environment in which all concerned can learn about, or *Appreciate*, and then describe the system of interest. Their task is to create an atmosphere in which all participants work towards a common understanding of the situation of interest, Vickers referred to this as “*Appreciation*” (Vickers 1968). The outcome of this cycle of learning is the consideration of ideas for *purposeful* action that might bring improvement to the situation – knowledge for action. The facilitator and participants conceptualise how the action might be put into practice and then consider the functional dimensions of the serving system. Knowledge for action is enriched as ideas are ‘contextualised’ to create a more detailed “complementary picture” (Vickers 1981). The outcome of this cycle of learning is to develop the sub-system structure so that the relevant properties of the system satisfies the *relationship maintaining* needs of the system as a whole. The participants can consider alternative strategies that will address their concerns.

It is important that a suitable way of undertaking such an inquiry is found that is within the soft paradigm. A proven framework to achieve this requirement is Soft Systems Methodology (SSM) (see Checkland and Poulter 2006). The development and application of SSM is well documented and unnecessary to rehearse its virtues here.^10^ Simply stated SSM is focused on developing an appreciation of the situation of interest, whose prime aim is to engender a cycle of learning. SSM begins by encouraging participants to consider the structure and processes that give the system of interest its form.

An alternative to SSM is the Appreciative Inquiry Method (Stowell 2012). In a paper entitled the Appreciative Inquiry Method (AIM) Stowell (2021a, b, c), traced the evolution of a soft method for knowledge elicitation, through to its use as a method of organisational inquiry.

AIM has shown itself to be valuable in situations where participants have reached an agreement about the issue to be addressed or a clear question to be investigated. While SSM is a powerful means of inquiring into ‘complex’ and ‘messy’ situations “…AIM is intended solely as a means of finding out what is considered to be the case in a given situation’ (West, 1995, p.144).

**Fig. 1:**
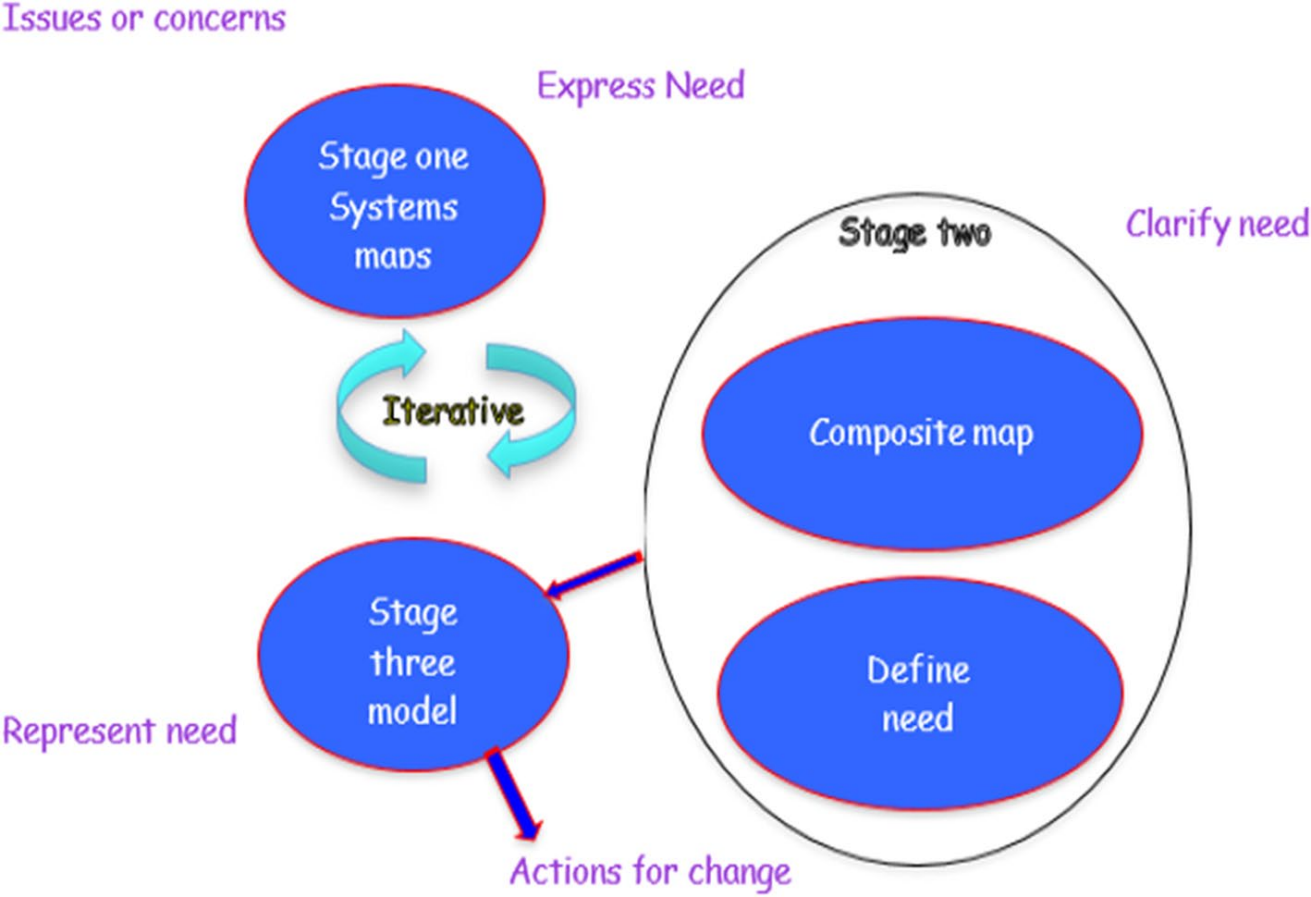
Schematic of AIM (Stowell 2021c)

As shown in Fig. 1 above, AIM consists of three stages and with each stage a practical commitment from participants. This activity takes the form of the production of a Systems Map which is a type of Venn diagram and, in this example, an activity model and influence diagram.

Stage one involves the participants being asked to produce individual maps around the agreed issue/question written in the centre of the diagram. Participants will then add their thoughts to the ellipses that, for them, make up the acknowledged issue. In some instances, this process can be described as an example of inductive^11^ logic as each participant draws conclusions from what *they* perceive the issues to be arising from the central question. It may be relevant or without foundation. Although these maps are personal opinions but when shared with other participants, they can provide a fruitful agenda for discussion within the group, particularly where there are differences of opinion.

Stage One and Two is concerned with the description of the map elements as purposeful activities. The expert is asked to describe each map element using PEArL (see below) to ensure that enough information is provided to develop the description of some purposeful human activity. With the help of the participants the facilitator will produce a single map, called a composite map inviting each participant to comment. In stage one the composite map consists of all the elements in each of the individual system maps, combining any areas of commonality. It should be noted that this stage is best undertaken with all participants as a means of engendering a debate and to improve group understanding. The facilitator seeks clarification about each sub-system. Experience has shown that the maps can be a mixture of what the participant thinks the situation to be and what they would like it to be. To address this Cooray (2010) found that by asking the client to describe the sub-system first in terms of *what is the case* (reality judgements), then *what ought* to be the case (‘judgements of the significance of these facts…’ to him/her and their society.. Vickers 1970, p.150) helps them to separate what is taking place presently and what they would like to happen.

The outcome of this stage is to discuss with the participants ways of combining individual group maps into a composite map; representing what the group as a whole think the situation to be; what is the case. This usually results in a debate and that helps the participants to gain a richer appreciation of the issue or question posed. Once there is a clear agreement of the makeup of the ‘issue’ then in Stage Two the participants repeat the above but this time the maps are based on what the participants would like the situation to be; what ought to be. As before after discussion the outcome is a composite systems map based upon ‘what ought’ to be the case.

Moving on to the second part of Stage Two the participants are asked to select one or all of the sub-systems and then define each subsystem, usually in the form of a description similar to the Root Definition stage of SSM. Once all participants have *authenticated* the definition (Champion and Stowell 2001p.27–28) the final task, Stage Three, is to produce a model of the system or systems that make up the collective view of the centre issue or question. It is not unusual for the model to be in the form of an activity model although other systems diagrams may be more appropriate (see Stowell and Welch, pp.25–31).

While AIM has shown its value in a workshop environment in the next section, I will discuss two projects where AIM was used in a virtual setting.

## Virtual Action Research for Virtual Organisations

In their paper Stowell and Cooray (2017) applied the method where face-to-face meetings were not possible because of the geographical separation between participants. The project was initially set up using email, but it became apparent to replicate the kind of environment that would take place in a face-to-face setting the participants needed to meet. To enable this an asynchronous ‘virtual environment’ was created which enabled participants to talk to each other and see the other participants in real time. This configuration provided a better balance between participants and provided a platform for equal involvement between the members. In order to simulate as much as possible a situation where all participants (both in the UK and the USA) felt part of the same process each member was provided with individual laptops with microphones and headsets; Access to a connection to the internet, the shared use of Wiggio, a virtual meeting software, delivered over the internet.

Once this set up was agreed the next task was to define the boundary, both physical and in terms of expertise, Stowell and Cooray also applied the mnemonic CATWOE (from SSM) (ibid, 2017, pp.132–133) and PEArL (Champion and Stowell 2001) which is another systemic tool used frequently with AIM. The mnemonic PEArL provides a way of thinking about the composition of the group. PEArL consists of five different elements (see Table 1 below).

**Table 1:** List of PEArL Elements

Elements of PEArL	Issues to reflect upon
P-Participants	Who is Involved in the activity, who is excluded and why? Why are they involved? What is their role in the activity?
E- Engagement	How are the participants involved? What methods are used to engage participants? What are the environmental influences in which an activity takes place?
A- Authority	Formal authority associated with activity. What are the environmental influences? What embedded authority do the tools for engagement have? Why were they chosen and what influences the outcomes?
r- relationships	What kind of informal power or commodities (Stowell 2014, Stowell and Welch 2012, pp.116–118) do people use to influence others (Examples include the use of gender, sociability, and verbal skills)
L- Learning	The theoretical and practical outcomes from the activity, judgments about how these were achieved and assessment about the ownership of outcomes

Table Reproduced from Stowell and Cooray 2017 p.125.

In addition to using PEArL to think about the composition of the group it was found helpful as the means of enabling participants to reflect and discuss various aspects of the situation. To encourage the participants to think outside the box they were asked to think about CATWOE and the five elements of PEArL in terms of first 'what is the case’ which helped them reflect on how the program was currently actioned then ‘what ought to be the case without concerns of costs or other considerations (an ideal) (Vickers 1983). By asking participants to reflect upon the issue in this way it surfaced the multiple views of the situation. They were able to deconstruct the discussion into aspects they considered significant which in turn provided better understanding of the reasoning behind their decisions (their ‘W’ for want of a better word) and enhanced client learning.

Stowell and Cooray concluded that overall the exercise demonstrated that even in a virtual setting AIM offered the possibility of undertaking ‘soft’ A/R. They say that the exercise demonstrated *‘…how participants in a virtual synchronous team were able to go through several cycles of action and reflection to arrive at a better understanding of the problem domain, information requirements and technology needs…*’ (ibid, 2017).

The experience gained from this study suggested that AIM could be used in a virtual setting where the ‘normal’ gathering of participants was not possible. In the next study we describe how AIM was used once more in a virtual setting by Kramarova (Kramarova and Stowell 2021a, b, c) but this time, because of the restrictions on face-to-face meetings resulting from the Covid-19 pandemic the whole exercise was conducted virtually with no ‘group’ meetings. This exercise offered the opportunity to ‘stress test’ the approach.

## Using AIM in a virtual setting to investigate the impact of food deserts^12^ in the local community

A previous review of the literature into the notion of food deserts by Kramarova ‘…*showed there to be a predominance of reductionist methods and a dearth of interpretivist research. Previous investigations had been mainly undertaken using mapping techniques or measuring areas at high risk or areas with limited or no access to fresh food in the cities (areas known as ‘food deserts’). The literature surfaced no universal definition of food deserts*^13^* and it is doubtful if the outcomes are easily translatable into local communities*….’ (ibid, 2021, p.3). To take this investigation further the researchers decided to engage with a representative group of local citizens and employ soft action research to gain understanding of what the impact might be of, so called ‘food deserts’. The practicality of this was challenging as the researchers had to contend with the restrictions of face-to-face (F2F) meetings created by the restrictions arising from the Covid 19 pandemic and at the same time maintain the fundamentals of the ideas behind the chosen method and adapt it to a ‘total’ virtual setting. The experience gained from the Stowell and Cooray project provided useful background to the thinking about how to proceed with this project.^14^

As the first stage of the field research undertaken by Kramarova had yielded some clear questions to be investigated it was decided that the second field research was suited to AIM. Plans designed to engage with local participants were confounded by the restrictions as free movement was stopped. To overcome the difficulty the research was redesigned by employing information and communication technology (for full details see Kramarova and Stowell 2021a, b, c). The consequence of this was, could ‘suitable’ participants be found who were prepared to be involved in this kind of approach.

Possibly because of the restrictions on travel, a small group of suitable and willing participants was found. These included two experts concerned with procurement and distribution of FFV, a retail manager, a representative from environmental health plus two representatives from the local community, one a retired food science teacher and one a single parent with links to the citizens advice bureau. Each participant was ‘met’ by the facilitator/researcher and the task explained and then invited to participate. Each individual confirmed their willingness to be involved. The account that follows describes the outcomes and experiences of applying AIM facilitated by Zoom, Padlet and email as the prime methods of communication.^15^

## Virtual A/R and outcomes

In order to replicate the A/R process ‘virtually’ each participant was contacted individually by Zoom and, after a brief introduction of how to draw a map, they were then asked to produce their map using Zoom and Whiteboard. Following the introduction, the facilitators camera and microphone were switched off. After a short interval the participant declared they had completed the task. The facilitator ‘returned’ and raised any points of clarification but offered no comment upon the content of the diagram. When all participants had completed this stage^16^ the maps were combined into a ‘Composite Map’, a single all-encompassing map. This map was then circulated via email and the facilitator discussed it with each participant in turn. Where there were syntactical differences the map was modified with the agreement of the participants, then the final iteration was circulated via email and agreed via Padlet asynchronously with the entire group. The end result of this part of the exercise was a map representing what they consider ‘*Is the case*’ (reality judgement).

**Fig. 2:**
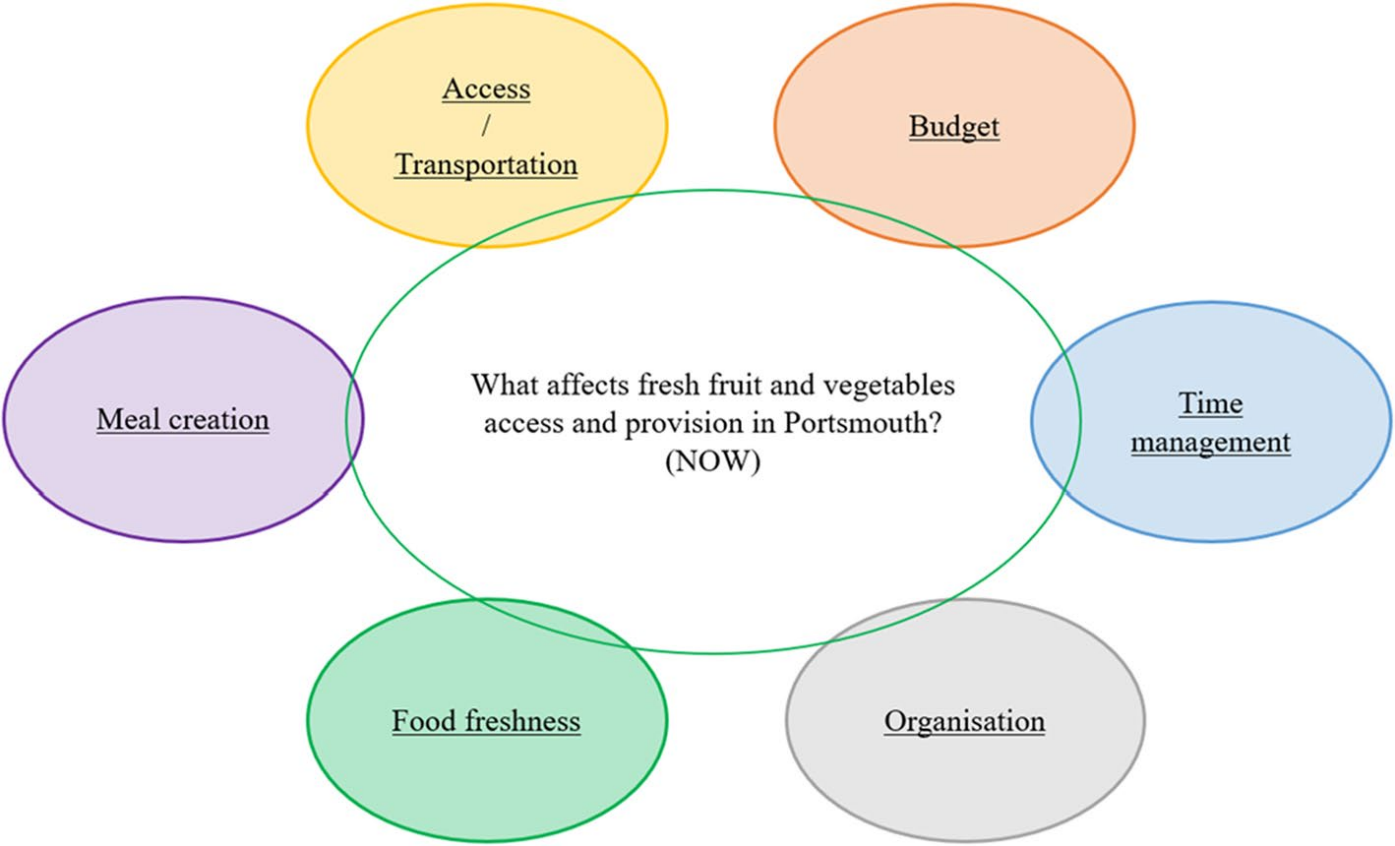
Agreed map of the current situation (what is the case)

This map was the result of a series of one-to-one meetings with the participants. The results at each stage was circulated via email and discussed individually via a Zoom link (see Kramarova and Stowell, 2021a, b, c). See Fig. 2 below.

By way of illustration the following table includes the comments that the participants made when discussing the map in Fig. 2 above.

**Fig. 3:**
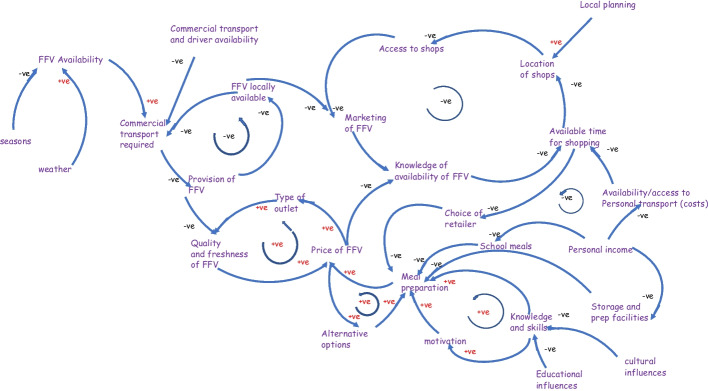
Influence diagram of the current situation (what is the case)

Table 2 above lists the kinds of challenges that the participants felt the local citizens faced. These comments provided the context of the map. In the next diagram, Fig. 3, an influence diagram, shows how the various factors in the whole process seemed to interrelate. It is worth noting that there are several positive feedback systems which will influence the way that the system as a whole operates. This diagram, which was developed by the authors and not the participants, could be developed further and provides the basis of a computer model using e.g. Vensim^17^. This kind of modelling would allow policy makers to assess the various impacts upon the system, as a whole, would have when adjusting different parts of the system.

**Table 2:** Providing a summary of the comments made by participants.

‘Name’ of sub-system	Summary of participants’ comments
Access / Transportation	Accessibility (e.g. distance, time, financial, health, availability in shop), convenience, transport links (e.g. household to gain fresh produce, depot to supplier, home deliveries), food waste
Budget (available)	Income, cost, price, cost compared to alternative options, food waste
Time management	Preparation, storage, household facilities, time, convenience, delays with supply (e.g. customs, weather)
Organising	Transport links, types of shops, locations, government initiatives, laws and regulations, retailer rents
Food freshness	Availability, seasons, weather, choice, quality, freshness, premium price, food waste
Meal creation	Education, culture, knowledge, understanding, nutritional skills, marketing, fast food options, comparative costs, school meals, choice of retailer, facilities for food storage and preparation, food waste

## What Ought to be the case

Following on from these meetings and the ‘what is the case’ map the next step was to move on to Stage Three. The exercise continued with the creation of individual maps as in Stages One and Two, but this time the participants were asked to draw maps of what they thought would be an ideal situation (what ought to be). Encouraging participants to reflect individually and as a virtual group on their answers to the central question, prompted their thinking process to appreciate the situation in terms of the ‘transformation’ that must take place to make the change from the current situation (what is the case) to describe ‘what ought to be the case’. This enabled the group to agree the six sub-systems as shown below.

**Fig. 4:**
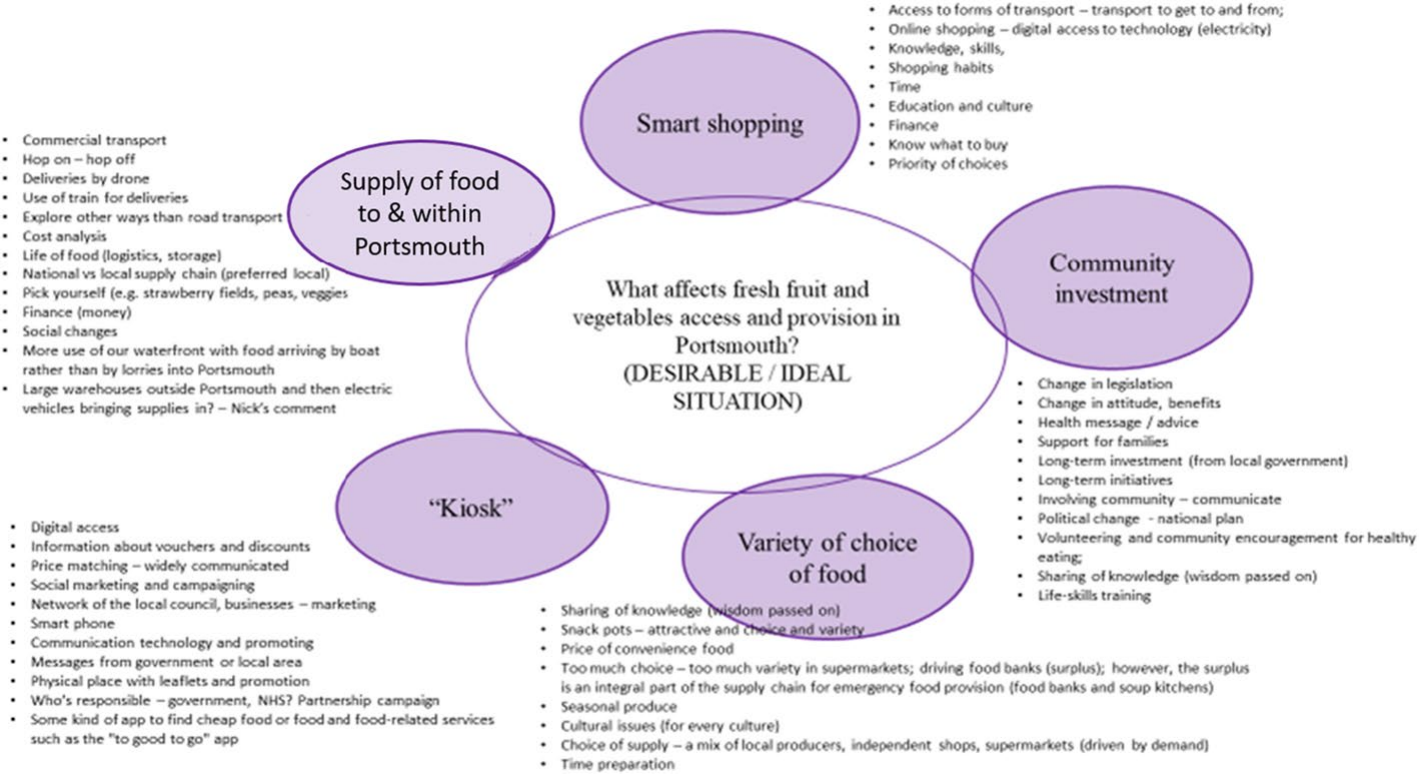
Agreed Map of what ought to be the case

The map below (Fig. 4) shows each of the areas thought by the group would address the question posed. Although an unnecessary addition to the diagrams we have included a summary of the comments made by the participants next to each sub system to provide the reader with insight into the discussions. Kramarova found these comments to be useful when considering the likely impact of implementing such a system.

## Actions for change

**Fig. 5:**
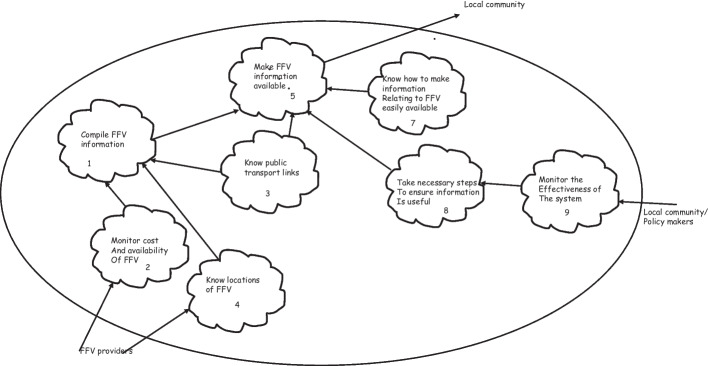
Activity Diagram of ‘Kiosk’ System

The outcome of reality and value judgements are a set of hypothetical relationships deemed significant enough to add/maintain/modify or delete in the given situation; An ‘action judgement’ follows where the feasibility of implementing the said relationships is explored. The main outcome of the appreciative cycle is learning that leads to a decision on action to be taken. As individuals make decisions and take action they learn from those actions, which in turn, cause their appreciative settings (standards, biases, values) to change. As appreciative settings of individuals change so do their future decisions, which are based on their altered appreciative settings. An activity model (e.g. see examples in Checkland and Poulter 2006, p.75), drawn by the authors, is shown in Fig. 5 below as an illustration of a way of representing the recommendations. We include the diagram to illustrate how the project might have progressed. The diagram shows the ‘real world impact’ and tasks that should be considered by policy makers when deliberating the recommendation for access and distribution of FFV in the Portsmouth area.

To complete the exercise an example of an activity model of one of the sub systems is given below as an illustration of representing the recommendations. I include them to illustrate how the project might have progressed. It should be noted that although these diagrams appear to be self-explanatory not everyone can understand them, although experiences of these models show this is unusual (for examples, see Checkland and Poulter 2006, pps.73; 118–119, Hart 2013, p.115) and I have found that most participants can quickly comprehend them.

Taking one of the sub-systems indicated in Fig. 4 above, Kiosk, can be defined as follows;

*A System to provide regularly refreshed and easily accessible information relating to the prices and availability of FFV, the locations and relevant transport links*

I represent this in the following activity model.

In spite of these diagrams being be self-explanatory not everyone can understand them, although experiences of these models show this is unusual (for examples, see Checkland and Poulter 2006, pps.73; 118–119, Hart 2013, p.115) and we have found that most participants can quickly comprehend them.

Taking one of the sub-systems indicated in Fig. 4 above, Kiosk, can be defined as follows;

A System to provide regularly refreshed and easily accessible information relating to the prices and availability of FFV, the locations and relevant transport links

This is represented in the following activity model.

The model above provides an illustration of how one of the named ‘systems’ could be activated and also serves as an agenda to discuss actions for change. Each of the ‘sub systems’ in the composite map can be modelled and provides the basis for a ‘system’ to address the issue/question explored. The next stage is to consider how each of the activities will be done. Examples of this process can be found in Stowell and West 1994, pp.174–193; Checkland and Poulter 2006, pp.118–121; Stowell and Welch 2012, pp.203–205.

## ‘Virtual soft action research’ -Learning from the practice^18^

### Setting the scene

The use of the FMA model (Checkland and Poulter 2006) helped to declare our frame of reference before the study began. Both studies reported here drew on the work of Checkland and the notion of recoverability (see Checkland 1981; Checkland and Poulter 2006), Champion and Stowell’s (2001) the notion of *authenticity*, and criteria for rigor in A/R (e.g. Iversen et al. 2004). These steps provided a third party with the opportunity to recover the process and follow the logical pathway that led to the conclusions drawn from the research.

Stowell and Cooray took care to record details of the study relating to the six criteria that Iversen et al. (2004) argues underpins the relevance and rigor in any A/R. They created PEArL records at the end of each session to reflect on and detail the way in which the researcher participated in the session*. ‘…Each record discussed the people the researcher engaged with (P), how they engaged with participants (E), who had the formal authority in each session (A), if the researcher was the initiator or recipient of informal authority (r), and what the researcher learnt about the way she engaged in each session (L). The process of reflection after each session helped the researcher to identify the ways in which she influenced the situation and take measures to address them. The records on the researcher’s interactions could also assist external parties to interpret results in the context of the situation…’* (ibid 2017).

We found that conducting a ‘virtual’ A/R to be considerably different to traditional A/R. It became clear in order to simulate a face-to-face meeting a means of setting up an asynchronous situation had to be found to help assure the quality of ‘virtual’ A/R practice, reporting and reviewing. While it is reasonable to assume these days that the majority of participants have a practical level of competence of using a variety of software, Kramarova found it helpful to give a brief introduction of how to use the method, with the chosen software. When selecting a communication tool, the researcher should consider one that has a shared drawing tool. Our research shows that having a shared visual representation of ideas helps reduce discussions becoming rambling, and it also helped to surface personal agendas.

### Systems diagrams and ideas

It is useful for any ‘soft’ systems researcher to have some appreciation/understanding of Soft systems ideas in general e.g. writing concise definitions and systems diagrams and they should recognize that not all participants will be aware of the kind of systems diagrams being used. It is helpful for the facilitator to provide an outline example of what is expected from each session, particularly in phase 1 and especially for phase 3. Soft systems ideas are not as easy to operationalise. For example, asking participants to consider the difference between *‘what is’* and *‘what ought to be the case’* has not always been easy to put across in an online inquiry.

Diagrams such as activity models might be puzzling even to academic participants or those new to ‘Systems’. Explaining the kind of outcomes and expectations for each session was found to be useful. Providing simple, easy to understand illustrations prior to each phase helps build confidence among the participants. This was particularly the case during Kramarova’s field research which was undertaken during the Covid-19 lockdown where each participant was working on their own. A brief introduction prior to the study, telling the participants, with simple examples, what is expected from them and provide a brief tutorial on how to use the adopted software proved to be helpful.^19^ We also found that a systems map could act as an agenda and was easy to understand and edit.

### Using the software

Undertaking synchronous and asynchronous ‘virtual A/R’ was fond to be significantly different to traditional A/R and adjustments to the way a virtual study was to be undertaken should be carefully considered as opportunities to quickly clarify issues are not so easily done as in a workshop setting. Those who claimed to be more technically experienced easily added, edited and deleted elements from the systems map. In the Stowell and Cooray project some participants professed to be less technically able and were reluctant to edit the map using the software tool. They frequently asked for help to complete the task rather than edit the map themselves. In such situations, they were asked for immediate feedback to ensure that these participants views were reflected in the resultant map. Since the meetings were held synchronously participants were able to provide instant feedback and make changes if the map didn’t reflect their views. In the Kramarova study the ease and ubiquity of the software tools did not present the participants with any similar difficulties.

### Boundary

Despite this being an online study, the importance of establishing its boundary became clear and careful consideration should be given to setting the study’s boundary at the start as it underpins the validity of its outcome. To provide rigour and establish the study’s ‘authenticity’, which means a formal declaration of the adopted approach and method (its conceptual framework) to the investigated issue, is declared before the investigation begins. (see Champion and Stowell 2001; Stowell and Welch 2012, p.180). Setting a boundary assists when considering the scope of the study and its limitations and helps to avoid any diversions to irrelevant areas/topics. A constant reflection on the boundary was found to be useful through the lens of the PEArL mnemonic^20^ (particularly P, E and A) at each of the AIM phases. Linkage to P, E and A enhances the clarity of initial stages of the inquiry as well as stressing the importance and identification of the boundary. The evolution of the boundary as the study proceeds is the result of the cycle of learning and is fundamental to soft A/R. The boundary must be flexible enough to enable and accommodate alterations as the inquiry proceeds, based on the learning and appreciation amongst all participants (Champion and Stowell 2001).

### Systems Tools

If ‘soft’ A/R is to be the basis of inquiry, then it is wise for the facilitators to decide the basis the participants could use for self-reflection and deconstruction of an issue of conflict *before* the project begins. For example, Stowell and Cooray discovered that the participants found questions corresponding to the elements from PEArL and CATWOE helped them organise their thoughts and explain their position in a structured way. This use of PEArL and CATWOE indirectly encouraged others to engage where there was a difference of opinion and was a way of creating shared understanding which helped reduce tensions and arrive at an agreement. Stowell and Cooray and Hart produced PEArL records in which they detailed the manner and atmosphere of each session This was done primarily to help external parties to interpret the results in the context in which the study took place. (Champion and Stowell 2001; Cooray 2010; Hart 2013).

### Conflict and Opinion

In an online meeting it may not always be possible to reflect upon the ‘messages’ that body language reveals that would be the case in face-to-face meetings. People tend to behave differently in an on-line setting and although the lack of human contact can, to a certain extent, be simulated via web cameras being on at all times, even then the facilitator has limited visibility of the participant and his/her space. Researchers should be aware of the difficulties of changing the original views of participants in virtual teams which makes agreeing an outcome harder to obtain. Virtual communications also raises the question of trust, whether the participants are alone, and if their performance is affected by something that is out of the facilitator’s sight. The impacts and its potential to mitigate outcomes of a virtual enquiry should be considered by the facilitator. Sarker and Valacich (2010) research suggests that conflict is more prevalent in virtual teams as participants are more unwilling to change their preconceptions and biases when they never or rarely meet face-to-face. In a report by Hancock and Woodworth (2012) cite Ducklow and Mortenson (2009) work where they say that *‘…When people are interacting face-to-face, there is something called the ‘motivational impairment effect,’ where your body will give off some cues as you become more nervous and there’s more at stake with your lie, In a computer-mediated environment, the exact opposite occurs.*^21^ (see report in Science Daily 2009).

Although most of the discussions in both studies were devoid of conflict, there were several conflictual situations in AIM cycles one and two. We found that initially participants were discussing the conflictual issues in an existential or high-level context rarely explaining the frame of mind or motivations for their assertions, which led to more accusations and tension. In order to reduce tension and focus the discussion around the specifics the researcher used the strategy of exploring the problem issue in the context. For Stowell and Cooray they found using the elements from PEArL and CATWOE (Checkland 1981) helped participants to draw attentions away from how they *felt emotionally* and identify underlying personal motivations for their views of the issue of conflict. This seemed a successful strategy as they then used the elements in PEArL and CATWOE to structure their assertions and present their reasoning in a more organized fashion (Stowell and Cooray 2017, p138).

Stowell and Cooray found that the communication software used, in their case Wiggio, hindered the authentication of the study. Traditionally when monitoring an A/R study the facilitator should be aware and record any use of ‘power’, they found PEArL to be a valuable aid in observing group interaction. For example, the facilitator could note (r) and the way in which participants engaged with each other (E). Although the software package allowed each participant to see and converse via the video feed to all other participants, they were confined visually to small windows in the software.^22^ We found that it was difficult for the researcher to monitor the many mini screens simultaneously leading to the possibility that many gestures and facial cues could be missed.

It is important that the facilitator keeps their involvement to a minimum because of the possibility that their presence might, consciously or subconsciously, influence the situation and the outcome (e.g. Galliers 1993; Gioia 1992). This leads us to question if the facilitator could truly observe and record the manner or atmosphere within which the synchronous virtual discussions were held which in turn compromises the authentication process of the study. The authentication process could be more difficult in asynchronous virtual communication since participants can add their contributions at different times limiting the facilitator’s ability to observe the manner/ atmosphere and group dynamics.

### Keeping to time

It is important to maintain a balance between encouraging rich discussions and keeping to time. In her pilot study Kramarova found time became an issue in all encounters and went beyond the estimates in the literature.

## Conclusion

As with all research and action research in particular the way that the research unfolds requires adjustment and flexibility. This is particularly the case with soft action research as the process itself is a cycle of learning about the situation and the approach. The key though is to maintain the underlying paradigm to prevent the research declining into anecdote. In these examples a method of soft action research was used as the vehicle for inquiry but used in a virtual environment where the interested parties could not meet. There were many lessons learnt but the main one is that undertaking soft action research can be accomplished between participants that are not gathered together in the same room as it the case in traditional action research. While there are disadvantages in not being in the same room this research opens up the possibility of undertaking a soft action research study with a greater number of participants covering a wide geographical area.

It is not claimed that these studies provide the definitive support for undertaking virtual action research, but the outcomes suggest that this is worth pursuing. As Stowell and Cooray point out.

‘…Virtual A/R subscribes to the same tenets as traditional AR in that theory and practice can be closely integrated by learning from the results of interventions that are planned after a thorough diagnosis of the problem domain’. (ibid, 2017, p.137).

## Footnotes

1 e.g. Checkland, (1999, pp.141–146).

2 The term Action Research is attributed to the work of Lewin 1948

3 Vickers, 1983(b), p.67 & c.4).

4 A recommended read for anyone interested in A/R.

5 See Husserl, 2012, pp.10–11; Majolino, 2015, pp.42–45 for further insight.

6 It is accepted that this is unlikely to be achieved but it should be the aim – a good example of this is Checkland’s exercise of producing an activity model of the fabricated definition of ‘bimps, bamps and bomps.

7 Dilthey (1991) distinguishes three classes of life-manifestations; Theoretical, Practical and Disclosive.

8 Adapted from Stowell, ‘Can Husserl’s Phenomenology Provide the Intellectual Framework of Soft Systems’ 2020.

9 Champion and Stowell 2003 and the notion of PEArL can be of value here

10 Checkland 1999; Checkland and Poulter 2006

11 In an inductive argument, the truth of the premises provides some degree of support for the truth of the conclusion. In deductive logic the premises of a valid deductive argument provide total support for the conclusion. See https://plato.stanford.edu/entries/logic-inductive/

12 The term is used as a means of identifying ‘populated urban areas with no access to healthy and affordable food’ (Beaumont et al., 1995 cited in Lu and Qiu, 2015; Cummins and Macintyre, 2002; Wrigley, 2002).

13 There are many ‘definitions’ of what Food desert means e.g. Cerovečki and Grünhagen, 2016; Sadler et al., 2015, but there is no commonly acceptable definition e.g. Cummins and Macintyre, 2002; Wrigley, 2002;

14 The way it was set up is described in an earlier paper by Kramarova and Stowell including the adaptation of the available technology (see Kramarova and Stowell, 2021a, b, c).

15 Wiggio that was used in Stowell and Cooray (2017) study no longer exists.

16 The elapsed for this stage of AIM was 3 weeks as meeting were dependent upon the availability of each participant.

17 https://vensim.com

18 Stowell and Cooray 2017

19 This is in cognizance of Stowell and Cooray’s observations where they observed that not all members of the group were prepared to use the technology see Stowell and Cooray (2016) Using Soft Systems Ideas within Virtual Teams.

20 Note: PEArL; P- Participants; E – Environment; A – Authority; r – relationships; L – Learning.

21 Ducklow and Mortenson, University of British Columbia. "Why People Are Better at Lying Online Than Telling A Lie Face-to-face." ScienceDaily. ScienceDaily, 5 May 2009. < www.sciencedaily.com/releases/2009/05/090503203738.htm > 

22 This was less of a problem for Kramarova with the software employed in the field study -see Kramarova and Stowell 2021a, b, c
